# A Treatable Cause of Pulsatile Tinnitus: A Case of Sigmoid Sinus Dehiscence

**DOI:** 10.7759/cureus.35577

**Published:** 2023-02-28

**Authors:** Abinayaa Purushothaman Ravichandran, Ye Su, Hussam A Yacoub

**Affiliations:** 1 Department of Neurology, Lehigh Valley Health Network, Allentown, USA

**Keywords:** sigmoid sinus dehiscence repair, cortical mastoidectomy, sigmoid sinus dehiscence, computed tomography of the temporal bone, headache, pulsatile tinnitus

## Abstract

Tinnitus is a symptom of an underlying condition that can be neurological, ontological, or infectious in origin. This case report describes a patient with pulsatile tinnitus caused by sigmoid sinus dehiscence, which was successfully treated by sigmoid sinus dehiscence repair. We recommend computed tomography angiography/magnetic resonance angiography or digital subtraction angiography to rule out vascular malformation, such as arteriovenous fistula, prior to surgical intervention. In addition, we recommend imaging of the brain and formal evaluation by an ophthalmologist and lumbar puncture prior to surgical intervention to rule out idiopathic intracranial hypertension if suspected.

## Introduction

Tinnitus is a perception of sound in proximity to the head in the absence of an external source. Tinnitus can be a symptom of an underlying condition that can be neurological, ontological, or infectious in origin. In pulsatile tinnitus (PT), the auditory perception is repetitively synchronous with the patient’s heartbeat. The underlying cause of PT is frequently determined and includes arterial, venous, or skull base disease [1].

Sigmoid sinus dehiscence (SSD) or sigmoid sinus diverticulum are commonly identified causes of PT, occurring in 25-35% of cases [2-4], and reported more frequently in women than men [4]. SSD is a rare condition in which there is a hole or dehiscence in the bone separating the sigmoid sinus from the middle ear. Alternatively, a diverticulum is an outpouching of the sinus itself. The most common symptoms of SSD include bone conduction hyperacusis, autophony, PT, and sound or pressure-induced vertigo. The SSD is typically diagnosed through imaging studies such as CT scans or MRIs. Treatment options include observation, hearing aids, or surgical repair of the dehiscence.

In addition, approximately half of the patients have SSD on the side of the dominant transverse sigmoid sinus due to the increased diameter and resultant turbulent flow [4]. Significant alleviation of the PT has been reported with gentle compression of the ipsilateral jugular vein [4].

## Case presentation

A 33-year-old woman presented to the hospital with a headache and symptoms of PT. The headache was in the right temporal region, rated as severe, and associated with the feeling of unsteadiness and a tendency to fall to the right. The PT in the right ear started eight days prior, with the gradual escalation of severity. Acetaminophen helped alleviate the headache but not the tinnitus. Nasal decongestants did not alleviate the symptoms. The tinnitus was significantly alleviated with compression of the right side of the neck. In addition, audiology testing completed by otolaryngology was unremarkable.

On initial examination, the patient was awake, alert, and oriented to person, place, and time. Funduscopic examination revealed no papilledema, and cranial nerve examination was unrevealing. Motor and sensory examinations were unremarkable. Reflexes, coordination, and gait examination were unrevealing. Laboratory workup, including complete blood count, comprehensive metabolic panel, and urinalysis, showed no abnormalities.

**Figure 1:**
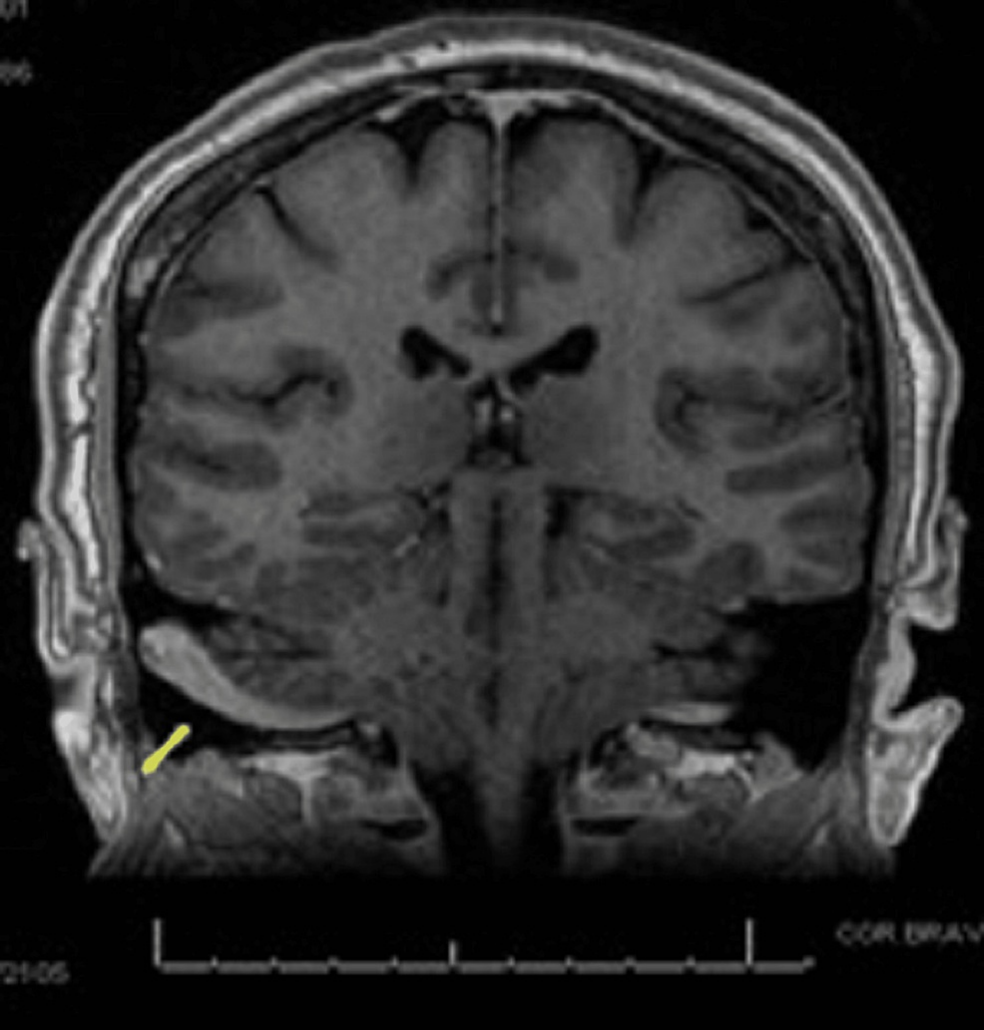
Coronal view of T1-weighted brain MRI revealing dominant right-sided sigmoid sinus (arrow) extending laterally toward the cortex of the mastoid bone.

**Figure 2:**
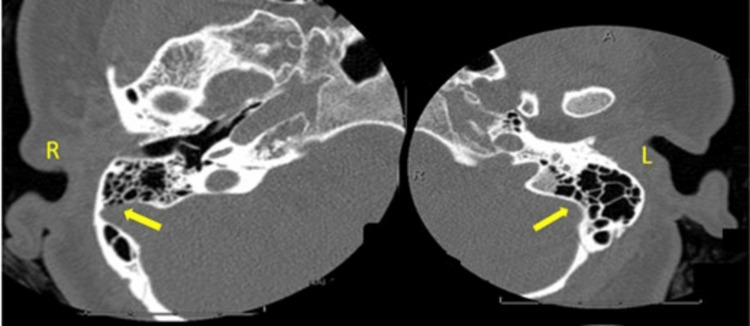
CT axial views revealing thinning, and an area of dehiscence, along the posterior wall of the right mastoid bone in close association with the sigmoid sinus compared to the left (arrows).

MRI of the brain showed an extension of the dominant right sigmoid sinus laterally toward the cortex of the mastoid segment of the temporal bone (Figure 1), raising the possibility of a dehiscent sigmoid sinus. A follow-up CT of the temporal bone revealed marked thinning and an area of dehiscence along the posterior wall of the right mastoid bone in close association with the right sigmoid sinus compared with the left (Figure 2).

The patient was evaluated by an otorhinolaryngologist for the imaging findings and referred for the repair of the skull malformation and build-up of bone overlying the sigmoid sinus. Three months following the initial presentation, the patient underwent a right-sided complete mastoidectomy and SSD repair. Following the procedure, the PT was completely resolved.

## Discussion

According to Sonmez et al. [5], the etiology of PT can be identified by imaging in approximately 67.6% of patients. If SSD is highly suspected in a patient with PT, CT of the temporal bones should be performed. CT studies should be interpreted cautiously, as false positives have been reported due to volume averaging [6]. MRI is warranted to rule out other etiologies, such as a space-occupying lesion or venous thrombosis. Opening pressure should be measured via lumbar puncture if idiopathic intracranial hypertension is suspected as the cause of PT.

SSD is effectively treated with cortical mastoidectomy and resurfacing of the dehiscence with an autogenous bone graft or bone cement and restores the normal separation between the sigmoid sinus and the middle ear. Wang et al. reported complete resolution of symptoms in approximately 74% of surgically treated patients, partial improvement in 14%, and no changes in 12% [4].

## Conclusions

This case report describes an important underlying treatable cause of PT, SSD, which can be treated with cortical mastoidectomy and resurfacing of the dehiscence with an autogenous bone graft or bone cement.
